# Supplementary material for: Heat adaptation of phage T7 under an extended genetic code
Source: Virus Evol. 2021 Dec 1;7(2):veab100. doi: 10.1093/ve/veab100 (PMC8923235; doi:10.1093/ve/veab100)
Supplement: veab100_Supp [file veab100_supp.zip › Sequencing_Analysis.zip!/Sequencing_Analysis/S3/evidence/RA_17.html]

BRESEQ :: Evidence


| Read alignment evidence... | | | | | | | | | | | |
| --- | --- | --- | --- | --- | --- | --- | --- | --- | --- | --- | --- |
|  | seq id | position | | ref | new | freq | score (cons/poly) | reads | annotation | genes | product |
| \* | NC\_001604.1 | 14,348 | 0 | T | C | 27.7% | 2286.8 / inf | 1813 | intergenic (–/–) | *–/–* | –/– |
| Reads supporting (aligned to +/- strand):  **ref** base T (630/670);  **new** base C (246/251);  **total** (883/930) | | | | | | | | | | | |
| Fisher's exact test for biased strand distribution *p*-value = 7.12e-01 | | | | | | | | | | | |
| Kolmogorov-Smirnov test that lower quality scores support variant *p*-value = 1.71e-02 | | | | | | | | | | | |

Only 100 of 1821 total aligned reads displayed.  

`GACAAGACAGACTGGCTGGAAGAAACCAGAAGTTACCAAGCTGAGTCATAAGGTGTTCGATAAGGACACTATGACCCACATCAAGGCTGGTGATTGGGTTAAGGTTGACATGGGAGTTGTTGGTGGATACGGCTACGTCCGCTCAGTTAGTGGCAAATATGCACAAGTGTCATACATCACAGGTGTTACTCCACGCGGTGCAATCGTTGCCGATAAGACCAACATGATTCACACAGGTTTCTTGACAGTTGTTTCATATGAAGAGATTGTTAAGTCACGATAATCAATAGGAGAAATCAATATGATCGTTTCTGACATCGAAGCTAACGCCCTCTTAGAGAGCGTCACTAAGTTCCACTGCGGGGTTATCTACGACTACTCCACCGCTGAGTACGTAAGCTACCGTCCGAGTGACTTCGGTGCGTATCTGGATGCGCTGGAAGCCGAGGTTGCACGAGGCGGTCTTATTGTGTTCCACAACGGTCACAAGTATGACGTTCCTGCATTGACCAAACTGGCAAAGTTGCAATTGAACCGAGAGTTCCACCTTCCTCGTGAGAACTGTATTGACACCCTTGTGTTGTCACGTTTGATTC  >  NC_001604.1/14052‑14647`  

                                                                                                                                                                                                                                                                                                        `|`                                                                                                                                                                                                                                                                                                             
`ttccagacgactacgagaagtttcgctggagagtcccattctaatacgactcactaaaggagacacaccatgttcaaactgattaagaagttaggccaactgctggttcgtatgtacaacgtggaagccaagcgactgaacgatgaggctcgtaaagaggccacacagtcacgcgctctggcgattcgctccaacgaactggctgacagtgcatccactaaagttaccgaggctgcccgtgtggcaaaccaagctcaacagctttccaaattctttgagtaatcaaacaggagaaaTcaaa                                                                                                                                                                                                                                                                                                         >  1:297562‑M2/297‑300 (MQ=255)`  
  `ccagacgactacgagaagtttcgctggagagtcccattctaatacgactcactaaaggagacacaccatgttcaaactgattaagaagttaggccaactgctggttcgtatgtacaacgtggaagccaagcgactgaacgatgaggctcgtaaagaggccacacagtcacgcgctctggcgattcactccaacgaactggctgacagtgcatccactaaagttaccgaggctgcccgtgtggcaaaccaagctcaacagctttccaaattctttgagtaatcaaacaggagaaaTCAatat                                                                                                                                                                                                                                                                                                       <  1:35310‑M2/7‑1 (MQ=255)`  
     `gacgcctacgagaagtttcgctggagggtcccattctaatacgactcactaaaggagacacaccatgttcaaactgattaagaagttaggccaactgctggttcgtatgtacaacgtggaagccaagcgactgaacgatgaggctcgtaaagaggccacacagtcacgcgctctggcgattcgctccaacgaactggctgacagtgcatccactaaagttaccgaggctgcccgtgtggcaaaccaagctcaacagctttccaaattctttgagtaatcaaacaggagaaaTCAATATGAt                                                                                                                                                                                                                                                                                                    <  1:100087‑M2/10‑1 (MQ=255)`  
     `gacgactacgagaagtttcgctggagagtcccattctaatacgactcactaaaggagacacaccatgttcaaactgattaagaagttaggccaactgctggttcgtatgtacaacgtggaagccaagcgactgaacgatgaggctcgtaaagaggccacacagtcacgcgctatggcgattcgctccaacgaactggctgacagtgcatccactaaagttaccgaggctgcccgtgtggcaaaccaagctcaacagctttccaaattctttgagtaatcaaacaggagaaaTCAATATGAt                                                                                                                                                                                                                                                                                                    <  1:343681‑M2/10‑1 (MQ=255)`  
                           `ggagagtcccattctaatacgactcactaaaggagacacaccatgttcaaactgattaagaagttaggccaactgctggttcgtatgtacaacgtggaagccaagcgactgaacgatgaggctcgtaaagaggccacacagtcacgcgctctggcgattcgctccaacgaactggctgacagtgcatccactaaagttaccgaggctgcccgtgtggcaaaccaagctcaacagctttccaaattctttgagtaatcaaacaggagaaaCCATTATGATCGTTTCTGACATCGAAGCTAAc                                                                                                                                                                                                                                                                              >  1:6747‑M2/270‑301 (MQ=255)`  
                                `gtcccattctaatacgactcactaaaggagacacaccatgttcaaactgattaagaagttaggccaactgctggttcgtatgtacaacgtggaagccaagcgactgaacgatgaggctcgtaaagaggccacacagtcacgcgctctggcgattcgctccaacgaactggctgacagtgcatccactaaagttaccgaggctgcccgtgtggcaaaccaagctcaacagctttccaaattctttgagtaatccaacaggagaaaTCAATATGATCGTTTCTGACATCGAAGCTAACgcccc                                                                                                                                                                                                                                                                         >  1:156131‑M2/265‑300 (MQ=255)`  
                                `gtcccattctaatacgactcactaaaggagacacaccatgttcaaactgattaagaagttaggccaactgctggttcgtatgtacaacgtggaagccaagcgactgaacgatgaggctcgtaaagaggccacacagtcacgcgctctggcgattcgctccaacgaactggctgacagtgcatccactaaagttaccgaggctgcccgtgtggcaaaccaagctcaacagctttccaaattctttgagtaatcaaacaggagaaaTCAATATGATCGTTTCTGACATCGAAGCTAACGCCct                                                                                                                                                                                                                                                                         >  1:161857‑M2/265‑301 (MQ=255)`  
                                     `attctaatacgactcactaaaggagacacaccatgttcaaactgattaagaagttaggccaactgctggttcgtatgtacaacgtggaagccaagcgactgaacgatgaggctcgtaaagaggccacacagtcacgcgctctggcgattcgctccaacgaactggctgacagtgcatccactaaagttaccgaggctgcccgtgtggcaaaccaagctcaacagctttccaaattctttgagtaatcaaacaggagaaaCCATTATGATCGTTTCTGACATCGAAGCTAACGCCCTCTTag                                                                                                                                                                                                                                                                    <  1:168121‑M2/42‑1 (MQ=255)`  
                                      `ttctaatacgactcactaaaggagacacaccatgttcaaactgattaagaagttaggccaactgctggttcgtatgtacaacgtggaagccaagcgactgaacgatgaggctcgtaaagaggccacacagtcacgtgctctggcgattcgctccaacgaactggctgacagtgcatccactaaagttaccgaggctgcccgtgtggcaaaccaagctcaacagctttccaaattctttgagtaatcaaacaggagaaaTCAATATGATCGTTTCTGACATCGAAGCTAACGCCCTCTTaga                                                                                                                                                                                                                                                                   >  1:309437‑M2/259‑301 (MQ=255)`  
                                      `ttctaatacgactcactaaaggagacacaccatgttcaaactgattaagaagttaggccaactgctggttcgtatgtacaacgtggaagccaagcgactgaacgatgaggctcgtaaagaggccacacagtcacgcgctctggcgattcgctccaacgaactggctgacagtgcatccactaaagttaccgaggctgcccgtgtggcaaaccaagctcaacagctttccaaattctttgagtaatcaaacaggagaaaTCAATATGATCGTTTCTGACATCGAAGCTAACGCCCTCTtagc                                                                                                                                                                                                                                                                   >  1:123885‑M2/259‑300 (MQ=255)`  
                                      `ttctaatacgactcactaaaggagacacaccatgttcaaactgattaagaagttaggccaactgctggttcgtatgtacaacgtggaagccaagcgactgaacgatgaggctcgtaaagaggccacacagtcacgcgctctggcgattcgctccaacgaactggctgacagtgcatccactaaagttaccgaggctgcccgtgtggcaaaccaagctcaacagctttccaaattctttgagtaatcaaacaggagaaaCCATTATGATCGTTTCTGACATCGAAGCTAACGCCCTCTTaga                                                                                                                                                                                                                                                                   >  1:142177‑M2/259‑301 (MQ=255)`  
                                       `ttataagagacagactaaaggcgacacaccatgttcaaactgattaagaagttaggccaactgctggttcgtatgtacaacgtggaagccaagcgactgaacgatgaggctcgtaaagaggccacacagtcacgcgctctggcgattcgctccaacgaactggctgacagtgcatccactaaagttaccgaggctgcccgtgtggcaaaccaagctcaacagctttccaaattctttgagtaatcaaacaggagaaaTCAATATGATCGTTTCTGACATCGAAGCTAACGCCCTCTTagag                                                                                                                                                                                                                                                                  <  1:328701‑M2/44‑1 (MQ=255)`  
                                       `tctaatacgactcactaaaggagacacaccatgttcaaactgattaagaagttaggccaactgctggttcgtatgtacaacgtggaagccaagcgactgaacgatgaggctcgtaaagaggccacacagtcacgcgctctggcgattcgctccaacgaactggctgacagtgcatccactaaagttaccgaggctgcccgtgtggcaaaccaagctcaacagctttccaaattctttgagtaatcaaacaggagaaaCCATTATGATCGTTTCTGACATCGAAGCTAACGCCCTCTTagag                                                                                                                                                                                                                                                                  <  1:81225‑M2/44‑1 (MQ=255)`  
                                       `agtaatacgactcactaaaggagacaaaccatgttcaaactgattaagaagttaggccaactgctggttcgtatgtacaacgtggaagccaagcgactgaacgatgaggctcgtaaagaggccacacagtcacgcgctctggcgattcgctccaacgaactggctgacagtgcatccactaaagttaccgaggctgcccgtgtggcaaaccaagctcaacagctttccaaattctttgagtaatcaaacaggagaaaCCATTATGATCGTTTCTGACATCGAAGCTAACGCCCTCTTagag                                                                                                                                                                                                                                                                  <  1:337920‑M2/44‑1 (MQ=255)`  
                                       `agatgtatataagagacggggagacacaccatgttcaaactgattaagaagttaggccaactgctggttcgtatgtacaacgtggaagccaagcgactgaacgatgaggctcgtaaagaggccacacagtcacgcgctctggcgattcgctccaacgaactggctgacagtgcatccactaaagttaccgaggctgcccgtgtggcaaaccaagctcaacagctttccaaattctttgagtaatcaaacaggagaaaTCAATATGATCGTTTCTGACATCGAAGCTAACGCCCTCTTagag                                                                                                                                                                                                                                                                  <  1:195210‑M2/44‑1 (MQ=255)`  
                                       `actaatacgactaactaaaggagacacaccaggttcaaactgattaagaagttaggccaactgctggttcgtatgtacaacgtggaagccaagcgactgaacgatgaggatcgtaaagaggccacacagtcacgcgctctggcgattcgctccaacgaactggctgacagtgcatccactaaagttaccgaggctgcccgtgtggcaaaccaagctcaacagctttccaaattctttgagtaatcaaacaggagaaaTCAATATGATCGTTTCTGACATCGAAGCTAACGCCCTCTTagag                                                                                                                                                                                                                                                                  <  1:243235‑M2/44‑1 (MQ=255)`  
                                        `ctaatacgactcactaaaggagacacaccatgttcaaactgattaagaagttaggccaactgctggttcgtatgtacaacgtggaagccaagcgactgaacgatgaggctcgtaaagaggccacacagtcacgcgctctggcgattcgctccaacgaactggctgacagtgcatccactaaagttaccgaggctgcccgtgtggcaaaccaagctcaacagctttccaaattctttgagtaatcaaacaggagaaaTCAATATGATCGTTTCTGACATCGAAGCTAACGCCCTCTTagaga                                                                                                                                                                                                                                                                 >  1:245527‑M2/257‑301 (MQ=255)`  
                                         `taatacgactcactaaaggagacacaccatgttcaaactgattaagaagttaggccaactgctggttcgtatgtacaacgtggaagccaagcgactgaacgatgaggctcgtaaagaggccacacagtcacgcgctctggcgattcgctcaaacgaactggctgacagtgcatccactaaagttaccgaggctgcccgtgtggcaaaccaagctcaacagctttccgaattctttgagtaatcaaacaggagaaaTCAATATGATCGTTTCTGACATCGAAGCTAACGCCCTCTTagagag                                                                                                                                                                                                                                                                <  1:200130‑M2/46‑1 (MQ=255)`  
                                         `ctaatacgactcactaaaggagacacaccatgttcaaactgattaagaagttaggccaactgctggttcgtatgtacaacgtggaagccaagcgactgaacgatgaggctcgtaaagaggccacacagtcacgcgctctggcgattcgctccaacgaactggctgacagtgcatccactaaagttaccgaggctgcccgtgtggcaaaccaagctcaacagctttccaaattctttgagtaatcaataggagaaaTCAATATGATCGTTTCTGACATCGAAGCTAACGCCCTCttagagct                                                                                                                                                                                                                                                                >  1:205067‑M2/256‑299 (MQ=255)`  
                                          `aatacgactcactaaaggagacacaccatgttcaaactgattaagaagttaggccaactgctggttcgtatgtacaacgtggaagccaagcgactgaacgatgagtctcgtaaagaggccacacagtcacgcgctctggcgattcgctccaacgaactggctgacagtgcatccactaaagttaccgaggctgcccgtgtggcaaaccaagctcaacagctttccaaattctttgagtaatcaaacaggagaaaCCATTATGATCGTTTCTGACATCGAAGCTAACGCCCTcttagagctg                                                                                                                                                                                                                                                               >  1:136266‑M2/255‑298 (MQ=255)`  
                                                 `ctcactaaaggagacacaccatgttcaaactgattaagaagttaggccaactgctggttcgtatgtacaacgtggaagccaagcgactgaacgatgaggctcgtaaagaggccacacagtcacgcgctctggcgattcgctccaacgaactggctgacagtgcatccactaaagttaccgaggctgcccgtgtggcaaaccaagctcaacagctttccacattctttgagtaatcaaacaggagaaaTCAATATGATCGTTTCTGACATCGAAGCTAacgccctcttagagctgtctcttt                                                                                                                                                                                                                                                        >  1:325103‑M2/248‑291 (MQ=255)`  
                                                     `ctaaaggagacacaccatgttcaaactgattaagaagttaggccaactgctggttcgtatgtacaacgtggaagccaagcgactgaacgatgaggctcgtaaagaggccacacagtcacgcgctctggcgattcgctccaacgaactggctgacagtgcatccactaaagttaccgaggctgcccgtgcggcaaaccaagcccaacagctttccaaattcttttagtaatctaacaggagaaaTCAATATGATCGTTTCTGACATCGAAGCTAACGCCCTCCTAGAGAGCGTCACTAAGtt                                                                                                                                                                                                                                                    >  1:13923‑M2/244‑301 (MQ=255)`  
                                                       `aaaggagacacaccatgttcaaactgattaagaagttaggccaactgctggttcgtatgtacaacgtggaagccaagcgactgaacgatgaggctcgtaaagaggccacacagtcacgcgctctggcgattcgctcccacgaactggctgacagtgcatccactaaagttaccgaggctgcccgtgtggcaaaccaagctcaacagctttccaaattctttgagtaataaaacaggagaaaCCATTATGATCGCTTCTGAACTCGAAGCTAACGCTCTCTTAGAGAGCGTCActaagcctg                                                                                                                                                                                                                                                  >  1:148795‑M2/242‑297 (MQ=255)`  
                                                         `aaggagacacaccatgttcaaactgattaagaagttaggccaactgctggttcgtatgtacaacgtggaagccaagcgactgaacgatgaggctcgtaaagaggccacacagtcacgcgctctggcgattcgctcaaacgaactggctgacagtgcatccactaaagttaccgaggctgcccgtgtggcaaaccaagctcaacagctttccaaattctttgagtaatcaataggagaaaTCAATATGATCGTTTCTGACATCGAAGCTAACGCCCTCTTAGAGAGCGTCACTAAGTTCCAc                                                                                                                                                                                                                                                <  1:30396‑M2/62‑1 (MQ=255)`  
                                                            `agacacaccatgttcaaactgattaagaagttaggccaactgctggttcgtatgtacaacgtggaagccaagcgactgaacgatgaggctcgtaaagaggccacacagtcacgcgctctggcgattcgctccaacgaactggctgacagtgcatccactaaagttaccgaggctgcccgtgtggcaaaccaagctcaacagctttccaaattctttgagtaatcaaacaggagaaaTCAATATGATCGTTTCTGACATCGAAGCTAACGCCCTCTTAGAGAGCGTCACTAAGTTCCACTGc                                                                                                                                                                                                                                             >  1:250865‑M2/237‑301 (MQ=255)`  
                                                                 `caccatgttcaaactgattaagaagttaggccaactgctggttcgtatgtacaacgtggaagccaagcgactgaacgatgaggctcgtaaagaggccacacagtcacgcgctctggcgattcgctccaacgaactggctgacagtgcatccactaaagttaccgaggctgcccgtgtggcaaaccaagctcaacagctttccaaattctttgagtaatcaaacaggagaaaTCAATATGATCGTTTCTGACATCGAAGCTAACGCCCTCTTAGAGAGCGTCACTAAGTTCCACTGCGGGGt                                                                                                                                                                                                                                        <  1:91619‑M2/70‑1 (MQ=255)`  
                                                                 `aaccatgttcaaactgattaagaagttaggccaactgctggttcgtatgtacaacgtggaagccaagcgactgaacgatgaggctcgtaaagaggccacacagtcacgcgctctggcgattcgctccaacgaactggctgacagtgcatccactaaagttgccgaggctgcccgtgtggcaaaccaagctcaacagctttccaaattctttgagtaatcaaacaggagaaaTCAATATGATCGTTTCTGACATCGAAGCTAACGCCCTCTTAGAGAGCGTCACTAAGTTCCACTGCGGGGt                                                                                                                                                                                                                                        <  1:62944‑M2/70‑1 (MQ=255)`  
                                                                      `tgttcaaactgattaagaagtttggccaactgctggttcgtatgtacaacgtggaagccaagcgactgaacgatgaggctcgtaaagaggccacacagtcacgcgctctggcgattcgctccaacgaactggctgacagtgcatccactaaagttaccgaggctgcccgtgtggcaaaccaagctcaacagctttccaaattctttgagtaatcaaacagtagaaaTCAATATGATCGTTTCTGACATCGAAGCTAACGCCCTCTTAGAGAGCGTCACTAAGTTCCACTGCGGGGTTATCt                                                                                                                                                                                                                                   >  1:257181‑M2/227‑301 (MQ=255)`  
                                                                      `tgttcaaactgattaagaagttaggccaactgctggttcgtatgtacaacgtgtaagccaagcgactgaacgatgaggctcgtaaagaggccacacagtcacgcgctctggcgattcgctccaacgaactgactgacagtgcatccactaaagttaccgaggctgcccgtgtggcaaaccaagctcaacagctttccaaattctttgagtaatcaaacaggagaaaCCATTATGATCGTTTCTGACATCGAAGCTAACGCCCTCTTAGAGAGCGTCACTAAGTTCCACTGCGGGGTTATCt                                                                                                                                                                                                                                   >  1:291988‑M2/227‑301 (MQ=255)`  
                                                                       `gttcaaactgattaagaagttaggccaactgctggttcgtatgtacaacgtggaagccaagcgactgaacgatgaggctcgtaaagaggccacacagtcacgcgctctggcgattcgctccaacgaactggctgacagtgcatccactaaagttaccgaggctgcccgtgtggcaaaccaagctcaacagctttccaaattctttgagtaatcaaacaggagaaaTCATTATGATCGTTTCTGACATCGAAGCTAACGCCCTCTTAGAGAGCGTCACTAAGTTCCACTGCGGGGTTATCTa                                                                                                                                                                                                                                  >  1:335316‑M2/226‑301 (MQ=255)`  
                                                                        `ttcaaactgattaagaagttaggccaactgctggttcgtatgtacaacgtggaagccaagcgactgaacgatgaggctcgtaaagaggccacacagtcacgcgctctggtgattcgctccaacgaactggctgacagtgcatccactaaagttaccgaggctgcccgtgtggcaaaccaagctcaacagctttccaaattctttgagtaatcaaacaggagaaaTCAATATGATCGTTTCTGACATCGAAGCTAACGCCCTCTTAGAGAGCGTCACTAAGTTCCACTGCGGGGTTATCTAc                                                                                                                                                                                                                                 <  1:77609‑M2/77‑1 (MQ=255)`  
                                                                           `aaactgattaagaagttaggccaactgctggttcgtatgtacaacgtggaagccaagcgactgaacgatgaggctcgtaaagaggccacacagtcgcgcgctctggcgattcgctccaacgaactggctgacagtgcatccactaaagttaccgaggctgcccgtgtggcaaaccaagctcaacagctttccaaattctttgagtaatcaaacaggagaaaTCAATATGATCGTTTCTGACATCGAAGCTAACGCCCTCTTAGAGAGCGTCACTAAGTTCCACTGCGGGGTTATCTACGac                                                                                                                                                                                                                              <  1:152708‑M2/80‑1 (MQ=255)`  
                                                                           `aaactgattaagaagttaggccaactgctggttcgtatgtacaacgtggaagccaagcgactgaacgatgaggctcgtaaagaggccacacagtcacgcgctctagcgattcgctccaacgaactggctgacagtgcatccactaaagttaccgaggctgcccgtgtggcaaaccaagctcaacagctttccaaattctttgagtaatcaaacaggagaaaCCATTATGATCGTTTCTGACATCGAAGCTAACGCCCTCTTAGAGAGCGTCACTAAGTTCCACTGCGGGGTTATCTACGac                                                                                                                                                                                                                              <  1:208922‑M2/80‑1 (MQ=255)`  
                                                                             `actgagtaagaagttaggccaactgctggttcgtatgtacaacgtggaagccaagcgactgaacgatgaggctcgtaaagaggccacacagtcacgcgctctggcgattcgctccaacgaactggctgacagtgcatccactaaagttaccgaggctgcccgtgtggcaaaccaagctcaacagctttccaaattctttgagtaatcaaacaggagaaaTCAATATGATCGTTTCTGACATCGAAGCTAACGCCCTCTTAGAGAGCGTCACTAAGTTCCACTTCGGGGTTATCTACGacta                                                                                                                                                                                                                            <  1:114016‑M2/82‑1 (MQ=255)`  
                                                                                   `aaagagacagggccaactgctggttcgtatgtacaacgtggaagccaagcgactgaacgatgaggctcgtaaagaggccacacagtcacgcgctctggcgattcgctccaacgaactggctgacagtgcatccactaaagttaccgaggctgcccgtgtggcaaaccaagctcaacagctttccaatttctttgagtaatcaaacaggagaaaTCAATATGATCGTTTCTGACATCGAAGCTAACGCCCTCTTAGAGAGCGTCACTAAGTTCCACTGCGGGGTTATCTACGACTACTCCAc                                                                                                                                                                                                                      <  1:37738‑M2/88‑1 (MQ=255)`  
                                                                                   `aaagaagttaggccaactgctggttcgtatgtacaacgtggaagcaaagcgactgaacgatgaggctcgtaaagaggccacacagtcacgcgctctggcgattcgctccaactaactggctgacagtgcatccactaaagttaccgaggcttcccgtgtggcaaaccaagctcaacagatttccaaattctttgagtaatcaaacaggagaaaTCAATATGATCGTTTCTGACATCGAAGCTAACGCCCTCTTAGAGAGCGTCACTAAGTTCCACTGCGGGGTTATCTACGACTACTCCAc                                                                                                                                                                                                                      <  1:70660‑M2/88‑1 (MQ=255)`  
                                                                                      `gaagttaggccaactgctggttcgtatgtacaacgtggaagccaagcgactgaacgatgaggctcgtaaagaggccacacagtcacgcgctctggcgattcgctccaacgaactggctgacagtgcatccactaaagttaccgaggctgcccgtgtggcaaaccaagctcaacagctttccaaattctttgagtaatcaaacaggagaaaTCAATATGATCTTTTCTGACATCGAAGCTAACGCCCTCTTAGAGAGCGTCACTAAGTTCCACTGCGGggttatctacgacctgtctcttat                                                                                                                                                                                                                   >  1:117947‑M2/211‑290 (MQ=255)`  
                                                                                      `gaagttaggccaactgctggttcgtatgtacaacgtggaagccaagcgactgaacgatgaggctcgtaaagaggccacacagtcacgcgctctggcgattcgctccaacgaactggctgacagtgcatccactaaagttaccgaggctgcacgtgtggcaaaccaagctcaacagctttccaaattctttgagtaatcaaacaggagaaaTCAATATGATCGTTTCTGACATCGAAGCAAACGCCCTCTTAGAGAGCGTTACTAAGTTCCACGGCGGGGTTATATACGACTACTCCACCGc                                                                                                                                                                                                                   >  1:104287‑M2/211‑301 (MQ=255)`  
                                                                                          `ttaggccaactgctggttcgtatgtacaacgtggaagccaagcgactgaacgatgaggctcgtaaagaggccacacagtcacgcgctctggcgattcgctccaacgaactggctgacagtgcatccactaaagttaccgaggctgcccgtgtggcaaaccaagctcaacagctttccaaattctttgagtaatcaaacaggagaaaTCAATATGATCGTTTCTGACATCGAAGCTAACGCCCTCTTAGAGAGCGTCACTAAGTTCCACTGCGGGGTTATCTACGACTACTCCACCGCTGAg                                                                                                                                                                                                               <  1:74698‑M2/95‑1 (MQ=255)`  
                                                                                           `taggccaagtgctggttcttatgtacaacgtggaatccaagcgactgaacgatgaggcgcgtaaagaggccacaaagtcacgcgctctggcgattcgctccaacgaactggctgacagtgcatccactaaagttaccgaggctgcccgtgtggcaaaccaagctcaacagctttccaaattctttgagtaatcaaacaggagaaaTCAATATGATCGTTTCTGACATCGAAGCTAACGCCCTCTTAGAGAGCGTCACTAAGTTCCACTGCGGGGTTATCTACGACTACTCCACCGCTGAGt                                                                                                                                                                                                              <  1:131284‑M2/96‑1 (MQ=255)`  
                                                                                             `agtcgacgtctgattcgtatgcaatacgtggaagccaagggcatgaacgatgaggcacgtaaagagtccacacagtcacgcggtctggcgattcgctccaacgaactggctgacagtgcatcgatgaaaagtactgaggctgtccgtgtggcaaaccaagctcaacagctttccaaattcttggagtaatcaaacaggataaaTCAATATGATCGTTTCTGACATCGTAGCTAACGCCCTCTTAGAGAGCGTCACTAAGTTCCACTTCTGGGTTATCTACGACTACTCCACCGCTGAGtat                                                                                                                                                                                                            <  1:176453‑M2/98‑2 (MQ=255)`  
                                                                                                  `actgctggttcgtatgcacaacgtggaagccaagcgactgaacgatgaggctcgtaaagaggccacacagtcacgcgctctggcgattcgctccaacgaactggctgacagtgcatccactaaagttaccgaggctgcccgtgtggcaaaccaagctcaacagctttccaaattctttgagtaatcaaacaggagaaaTCAATATGATCGTTTCTGACATCGAAGCTAACGCCCTCTTAGAGAGCGTCACTAAGTTCCACTGCGGGGTTATCTACGACTACTCCACCGCTGAGTACGTAAg                                                                                                                                                                                                       <  1:312441‑M2/103‑1 (MQ=255)`  
                                                                                                   `ctgctggttcgtatgtacaacgtggaagccaagcgactgaacgatgaggctcgtaaagaggccacacagtcacgcgctctggcgattcgatccaacgaactggctgacagtgcatccactaaagttaccgaggctgcccgtgtggcaaaccaagctcaacagctttccaaattctttgagtaatcaaacaggagaaaTCAATATGATCGTTTCTGACATCGAAGCTAACGCCCTCTTAGAGAGCGTCACTAAGTTCCACTGCGGGGTTATCTACGACTACTCCACCGCTGAGTACGTAAGc                                                                                                                                                                                                      <  1:109961‑M2/104‑1 (MQ=255)`  
                                                                                                    `ctgctggttcgtatgtacaacgtggaagccaagcgactgaacgatgaggctcgtaaagaggccacacagtcacgcgctctggcgattcgctccaacgaactggctgacagtgcatccactaaagttaccgaggctgccgtgtggcaaaccaagctcaacagctttccaaattctttgagtaatcaaacaggagaaaCCATTATGATCGTTTCTGACATCGAAGCTAACGCCCTCTTAGAGAGCGTCACTAAGTTCCACTGCGGGGTTATCTACGACTACTCCACCGCTGAGTACGTAAGCt                                                                                                                                                                                                     >  1:49365‑M2/197‑301 (MQ=255)`  
                                                                                                        `ggttcgtaggtacaacgtggaagccaagcgactgaacgatgaggctcgtaaagaggccacacagtcacgcgctctggcgattcgctccaacgaactggctgacagtgcatccactaaagttaccgaggctgcccgtgtggcaaaccaagctcaacagctttccaaattctttgagtaatcaaacaggagaaaCCATTATGATCGTTTCTGACATCGAAGCTAACGCCCTCTTAGAGAGCGTCACTAAGTTCCACTGCGGGGTTATCTACGACTACTCCACCGCTGAGTACGTAAGCTACCg                                                                                                                                                                                                 <  1:266860‑M2/109‑1 (MQ=255)`  
                                                                                                          `ttcgtatgtacaacgtggaagccaagcgactgaacgatgaggctcgtaaagaggccacacagtcacgcgctctggcgattcgctccaacgaactggctgacagtgcatccactaaagttaccgaggctgcccgtgtggcaaaccaagctcaacagctttccaaattctttgagtaatcaaacaggagaaaTCAATATGATCGTTTCTGACATCGAAGCTAACGCCCTCTTAGAGAGCGTCACTAAGTTCCACTGCGGGGTTATCTACGACTACTCCACCGCTGAGTCCGTAAGCTACCGTc                                                                                                                                                                                               >  1:270154‑M2/191‑301 (MQ=255)`  
                                                                                                          `ttcgtatgtacaacgtggaagccaagcgactgaacgatgaggctcgtaaagaggccacacagtcacgcgctctggcgattcgctccaacgaactggctgacagtgcatccactaaagttaccgaggctgcccgtgtggcaaaccaagctcaacagctttccaaattctttgagtaatcaaacaggagaaaTCAATATGATCGTTTCTGACATCGAAGCTAACGCCCTCTTAGAGAGCGTCACTAAGTTCCACTGCGGGGTTATCTACGACTACTCCACCGCTGAGTACGTAAGCTACCGTc                                                                                                                                                                                               <  1:233098‑M2/111‑1 (MQ=255)`  
                                                                                                            `cgtatgtacaacgtggaagccaagcgactgaacgatgaggctcgtaaagaggccacacagtcacgcgctctggcgattcgctccaacgaactggctgacagtgcatccactaaagttaccgagtctgcccgtgtggcaaaccaagctcaacagctttccacattctttgagtaatcaaacaggagaaaTCAATATGATCGTTTCTGACATCGAAGCTAACGCCCTCTTAGAGAGCGTCACTAAGTTCCACTGCGGGGTTATCTACGACTCCTCCACCTCTGAGTACGTAAGCTACCGTCCg                                                                                                                                                                                             >  1:227295‑M2/189‑301 (MQ=255)`  
                                                                                                            `cgtatgtacaacgtggaagccaagcgactgaacgatgaggctcgtaaagaggccacacagtcacgcgctctggcgattcgctccaacgaactggctgacagtgcatccactaaagttaccgaggctgcccgtgtggcaaaccaagctcaacagctttccaaattctttgagtaatcaaacaggagaaaTCAATATGATCGTTTCTGACATCGAAGCTAACGCCCTCTTAGAGAGCGTCACTAAGTTCCACTGCGGGGTTATCTACGACTACTCCACCGCTGAGTACGTAAGCTACCGTCCg                                                                                                                                                                                             >  1:1708‑M2/189‑301 (MQ=255)`  
                                                                                                                 `gtacaacgtggaagccaagcgactgaacgatgaggctcgtaaagaggccacacagtcacgcgctctggcgattcgctccaacgaactggctgacagtgcatccactaaagttaccgaggctgcccgtgtggcaaaccaagctcaacagctttccaaattctttgagtaatcaaacaggagaaaCCATTATGATCGTTTCTGACATCGAAGCTAACGCCCTCTTAGAGAGCGTCACTAAGTTCCACTGCGGGGTTATCTACGACTACTCCACCGCTGAGTACGTAAGCTACCGTCCGAGTGa                                                                                                                                                                                        >  1:107419‑M2/184‑301 (MQ=255)`  
                                                                                                                  `tacaacgtggaagccaagcgactgaacgatgaggctcgtaaagaggccacacagtcacgcgctctggcgattcgctccaacgaactggctgacagtgcatccactaaagttaccgaggctgcccgtgtggcaaaccaagctcaacagctttccaaattctttgagtaatcaaacaggagaaaTCAATATGATCGTTTCTGACATCGAAGCTAACGCCCTCTTAGAGAGCGTCACTAAGTTCCACTGCGGGGTTATCTACGACTACTCCACTGCTGAGTACGTAAGCTACCGTCCGAGTGAc                                                                                                                                                                                       >  1:180264‑M2/183‑301 (MQ=255)`  
                                                                                                                    `caacgtggaagccaagcgactgaacgatgaggctcgtaaagaggccacacagtcacgcgctctggcgattcgctccaacgaactggctgacagtgcatccactaaagttaccgaggctgcccgtgtggcaaaccaagctcaacagctttccaaattctttgagtaatcaaacaggagaaaTCAATATGATCGTTTCTGACATCGAAGCTAACGCCCTCTTAGAGAGCGTCACTAAGTTCCACTGCGGGGTTATCTACGACTACTCCACCGCTGAGTACGTAAGCTACCGTCCGAGTGACtt                                                                                                                                                                                     <  1:183661‑M2/121‑1 (MQ=255)`  
                                                                                                                                `caagcgacggaacgatgaggatcgtaaagaggccacacagtcacgcgctcgggcgattcgctccaacgaactggctgacagtgcatccactaaagttaccgaggctgcccgtgtggcaaacaaagctcaacagctgtccaaattctttgagtaatcaaacaggagaaaCCATTATGATCGTTTCTGACATCGAAGCTAACGCCCTCTTAGAGAGCGTCACTAAGTTCCACTGCGGGGTTATCTACGACTACTCCACCGCTGAGTACGTAAGCTACCGTCCGAGTGACTTCGGTGCGTATCt                                                                                                                                                                         <  1:230552‑M2/133‑1 (MQ=255)`  
                                                                                                                                                   `gctcgtaaagaggccacacagtcacgcgctctggcgattcgctccaacgaactggctgacagtgcatccactaaagttaccgaggctgcccgtgtggcaaaccaagctcaacagctttccaaattctttgagtaatcaaacaggagaaaCCATTATGATCGTTTCTGACATCGAAGCTAACGCCCTCTTAGAGAGCGTCACTAAGTTCCACTGCGGGGTTATCTACGACTACTCCACCGCTGAGTACGTAAGCTACCGTCCGAGTGACTTCGGTGCGTATCTGGATGCGCTGGAAGCCGAg                                                                                                                                                      >  1:223252‑M2/150‑301 (MQ=255)`  
                                                                                                                                                      `cgtaaagaggccacacagtcacgcgctctggcgattcgctccaacgaactggctgacagtgcatccactaaagttaccgaggctgcccgtgtggcaaaccaagctcaacagctttccaaattctttgagtaatcaaacaggagaaaTCAATATGATCGTTTCTGACATCGAAGCTAACGCCCTCTTAGAGAGCGTCACTAAGTTCCACTGCGGGGTTATCTACGACTACTCCACCGCTGAGTACGTAAGCTACCGTCCGAGTGACTTCGGTGCGTATCTGGATTCGCTGGAagcagagctg                                                                                                                                                   >  1:83493‑M2/147‑298 (MQ=255)`  
                                                                                                                                                           `agaggccacacagtcacgcgctctgtcgattcgctccaacgaactggctgacagtgcatccactaaagttaccgaggctgcccgtgtggcaaaccaagctcaacagctttccaaattctttgagtaatcaaacaggagaaaTCAATATGATCGTTTCTGACATCGAAGCTAACGCCCTCTTAGAGAGCGTCACTAAGTTCCACTGCGGGGTTATCTACGACTACTCCACCGCTGAGTACGTAAGCTACCGTCCGAGTGACTTCGGTGCGTATCTGGATGCGCTGGAAGCCGAGGTTGCACg                                                                                                                                              <  1:133969‑M2/160‑1 (MQ=255)`  
                                                                                                                                                            `gaggccacacagtcacgcgctctggcgattcgctccaacgaactggctgacagtgcatccactaaagttaccgaggctgcccgtgtggcaaaccaagctcaacagctttccaaattctttgagtaatcaaacaggagaaaTCAATATGATCGTTTCTGACATCGAAGCTAACGCCCTCTTAGAGAGCGTCACTAAGTTCCACTGCGGGGTTATCTACGACTACTCCACCGCTGAGTACGTAAGCTACCGTCCGAGTGACTTCGGTGCGTATCTGGATGCGCTGGAAGCCGAGGTTGCAcgg                                                                                                                                             >  1:300704‑M2/141‑300 (MQ=255)`  
                                                                                                                                                            `gaggccacacagtcacgcgctctggcgattcgctccaacgaactggctgacagtgcatccactaaagttaccgaggctgcccgtgtggcaaaccaagctcaacagctttccaaattctttgagtaatcaaacaggagaaaTCAATATGATCGTTTCTGACATCGAAGCTAACGCCCTCTTAGAGAGCGTCACTAAGTTCCACTGCGGGGTTATCTACGACTACTCCACCGCTGAGTACGTAAGCTACCGTCCGAGTGACTTCGGTGCGTATCTGGATGCGCTGGAAGCCGAGGTTGCACGa                                                                                                                                             <  1:321395‑M2/161‑1 (MQ=255)`  
                                                                                                                                                             `aggccacacagtcacgcgctctggcgattcgctccaacgaactggctgacagtgcatccactaaagttaccgaggctgcccgtgtggcaaaccaagctcaacagctttccaaattctttgagtaatcaaacaggagaaaTCAATATGATCGTTTCTGACATCGAAGCTAACGCCCTCTTAGAGAGCGTCACTAAGTTCCACTGCGGGGTTATCTACGACTACTCCACCGCTGAGTACGTAAGCTACCGTCCGAGTGACTTCGGTGCGTATCTGGATGCGCTGGCAGCCGAGGTTGCACGAg                                                                                                                                            >  1:59493‑M2/140‑301 (MQ=255)`  
                                                                                                                                                             `aggccacacagtcacgcgctctggcgattcgctccaacgaactggctgacagtgcatccactaaagttaccgaggctgcccgtgtggcaaaccaagctcaacagctttccaaattctttgagtaatcaaacaggagaaaTCAATATGATCGTTTCTGACATCGAAGCTAACGCCCTCTTAGAGAGCGTCACTAAGTTCCACTGCGGGGTTATCTACGACTACTCCACCGCTGAGTACGTAAGCTACCGTCCGAGTGAC‑TCGGTGCGTATCTGGATGCGctggaagccgagctgtctcttat                                                                                                                                           >  1:294596‑M2/140‑290 (MQ=255)`  
                                                                                                                                                              `ggccacacagtaacggggtctggcgattcgctccaacgaactggctgacagtgcatccactaaagttaccgaggctgcccgtgtggcaaaccaagctcaacagctttccaaattcgttgagtaatcaaacaggagaaaTCAATATGATCGTTTCTGACATCGGAGCTAACGCCCTCTTAGAGAGCGTCACTAAGTGCCACTGCGGGGTTATCTACGACTACTCCACCGCTGAGTACGTAAGCTACCGTCCGAGTGACTTCGGTGCGTATCTGGATGCGCTGGAAGCCGAGGTTGCACGAgg                                                                                                                                           <  1:31859‑M2/163‑1 (MQ=255)`  
                                                                                                                                                               `gccacacagtcacgcactctggcgattcgctccaacgaactggctgacagtgcatccactaaagttaccgaggctgcccgtgtggcaaaccaagctcaacagctttccaaattctttgagtaatcaaacaggagaaaCCATTATGATCGTTTCTGACATCGAAGCTAACGCCCTCTTAGAGAGCGTCACTAAGTTCCACTGCGGGGTTATCTACGACTACTCCACCGCTGAGTACGTAAGCTACCGTCCGAGTGACTTCGGTGCGTATCTGGATGCGCTGGAAGCCGAGGTTGCACGAGGc                                                                                                                                          <  1:57062‑M2/164‑1 (MQ=255)`  
                                                                                                                                                                       `gtcacgcgctctggcgattcgctccaacgaactggctgacagtgcatccactaaagttaccgaggctgcccgtgtggcaaaccaagctcaacagctttccaaattctttgagtaatcaaacaggagaaaTCAATATGATCGTTTCTGACATCGAAGCTAACGCCCTCTTAGAGAGCGTCACTAAGTTCCACTGCGGGGTTATCTACGACTACTCCACCGCTGAGTACGTAAGCTACCGTCCGAGTGACTTCGGTGCGTATCTGGATGCGCTGGAAGCCGAGGTTGCACGAGGCGGTCTTAt                                                                                                                                  >  1:288116‑M2/130‑301 (MQ=255)`  
                                                                                                                                                                         `cacgcgctctggcgattcgctccaacgaactggctgacagtgcatccactaaagttaccgaggctgcccgtgtggcaaaccaagctcaacagctttccaaattctttgagtaatcaaacaggagaaaTCAATATGATCGTTTCTGACATTGAAGCTAACGCCCTCTTAGAGAGCGTCACTAAGTTCCACTGCGGGGTTATCTACGACTACTCCACCGCTGAGTACGTAAGCTACCGTCCGAGTGACTTCGGTGCGTATCTGGATGCGCTGGAAGCCGAGGTTGCACGAGGCGGTCTTATtg                                                                                                                                <  1:281409‑M2/174‑1 (MQ=255)`  
                                                                                                                                                                         `cacgcgctctggcgattcgctccaacgaactggctgacagtgcatccactaaagttaccgaggctgcccgtgtggcaaaccaagctcaacagctttccaaattctttgagtaatcaaacaggagaaaTCAATATGATCGTTTCTGACATCGAAGCTAACGCCCTCTTAGAGAGCGTCACTAAGTTCCACTGCGGGGTTATCTACGACTACTCCACCGCTGAGTACGTAAGCTACCGTCCGAGTGACTTCGGTGCGTATCTGGATGCGCTGGAAGCCGAGGTTGCACGAGGCGGTCTTATtg                                                                                                                                <  1:86934‑M2/174‑1 (MQ=255)`  
                                                                                                                                                                         `cacgcgctctggcgattcgctccaacgaactggctgacagtgcatccactaaagttaccgaggctgcccgtgtggcaaaccaagctcaacagctttccaaattctttgagtaatcaaacaggagaaaTCAATATGATCGTTTCTGACATCGAAGCTAACGCCCTCTTAGAGAGCGTCACTAAGTTCCACTGCGGGGTTATCTACGACTACTCCACCGCTGAGTACGTAAGCTACCGTCCGAGTGACTTCGGTGCGTATCTGGATGCGCTGGAAGCCGAGGTTGCACGAGGCGGTCTTATtg                                                                                                                                <  1:202547‑M2/174‑1 (MQ=255)`  
                                                                                                                                                                         `cacgcgctctggcgattcgctccaacgaactggctgacagtgcatccactaaagttaccgaggctgcccgtgtggcaaaccaagctcaacagctttccaaattctttgagtaatcaaacaggagaaaCCATTATGATCGTTTCTGACATCGAAGCTAACGCCCTCTTAGAGAGCGTCACTAAGTTCCACTGCGGGGTTATCTACGACTACTCCACCGCTGAGTACGTAAGCTACCGTCCGAGTGACTTCGTTGCGTATCTGGATGCGCTGGAACCCGGGGTTGCACGGGGCGGTCtttttt                                                                                                                                >  1:172980‑M2/128‑300 (MQ=255)`  
                                                                                                                                                                         `cacgcgatctggtgattcgctccaacgaactggctgactgtgcatccactaaagttaccgatgcaccccgtgtggcaaaccaagctcaacagctttccaaattctgtgagtaatcaaacaggagaaaCCATTATGATCGTTTCTGACATCGAAGCTAACGCCCCCTGAGAGAGCGTCACTAAGTTCCACTGCGGGGTTATCTACGACTACTCCACCGCTGAGTACGGAAGCTACCGTCCGAGTGACTTCGGTGCGTATCTGGATGCGCTGGAAGCCGAGGTTGCACGAGGCGGTCTTATtg                                                                                                                                <  1:145856‑M2/174‑1 (MQ=255)`  
                                                                                                                                                                               `ctctggcgattcgctccaacgaactggctgacagtgcatccactaaagttaccgaggctgcccgtgtggcaaaccaagctcaacagctttccaaattctttgagtaatcaaacaggagaaaTCAATATGATCGTTTCTGACATCGAAGCTAACGCCCTCTTAGAGAGCGTCACTAAGTTCCACTGCGGGGTTATCTACGACTACTCCACCGCTGAGTACGTAAGCTACCGTCCGAGTGACTTCGGTGCGTATCTGGATGCGCTGGAAGCCGAGGTTGCacgaggcggtctctgtctcttat                                                                                                                          >  1:273521‑M2/122‑290 (MQ=255)`  
                                                                                                                                                                                             `cccaacgaactggctgacagtgcatccactaaagttaccgaggctgcccgtgtggaaaaccaagctcaacagctttccaaattctttgagtaatcaaacaggagaaaTCAATATGATCGTTTCTGACATCGAAGCTAACGCCCTCTTAGAGAGCGTCACTAAGTTCCACTGCGGGGTTATCTACGACTACTCCACCGCTGAGTACGTAAGCTACCGTCCGAGTGACTTCGGTGCGTATCTGGATGCGCTGGAAGCCGAGGTTGCACGAGGCGGTCTTATTGTGTTCCACAACGGTCACAAg                                                                                                            <  1:53825‑M2/194‑1 (MQ=255)`  
                                                                                                                                                                                                     `actggctgaaagggaatccactaaagttaccgaggctgcccgtgtggcaaaccaagctcaacagctttacaaattctttgagtaatcaaacaggagaaaCCATTATGATCGTTTCTGACATCGAAGCTAACGACCTCGTAGAGAGCGTCACAAAGTTCCACTGCGGGGTTATCTACGACTACTCCACCGCTGAGTACGTAAGCTACCGTCCGAGTGACTTCGGTGCGTATCTGGATGCGCTGGAAGCCGAGGTTGCACGAGGCGGTCTTATTGTGTTCCACAACGGTCACAAGTATGACGt                                                                                                    <  1:187863‑M2/202‑1 (MQ=255)`  
                                                                                                                                                                                                                      `ccactaaagttaccgaggctgcccgtgtggcaaaccaagctcaacagctttccaaattctttgagtaatcaaacaggagaaaTCAATATGATCGTTTCTGACATCGAAGCTAACGCCCTCTTAGAGAGCGTCACTAAGTTCCACTGCGGGGTTATCTACGACTACTCCACCGCTGAGTACGTAAGCTACCGTCCGAGTGACTTCGGTGCGTATCTGGATGCGCTGGAAGCCGAGGTTGCACGAGGCGGTCTTATTGTGTTCCACAACGGTCACAAGTATGACGTTCCTGCATTGaccactg                                                                                   >  1:262190‑M2/83‑298 (MQ=255)`  
                                                                                                                                                                                                                      `ccactaaagttaccgaggctgcccgtgtggcaaaccaagctcaacagctttccaaattctttgagtaatcaaacaggagaaaTCAATATGATCGTTTCTGACATCGAAGCTAACGCCCTCTTAGAGAGCGTCACTAAGTTCCACTGCGGGGTTATCTACGACTACTCCACCGCTGAGTACGTAAGCTACCGTCCGAGTGACTTCGGTGCGTATCTGGATGCGCTGGAAGCCGAGGTTGCACGAGGCGGTCTTATTGTGTTCCACAACGGTCACAAGTATGACGTTCCTGCATTGACCAAAc                                                                                   >  1:306745‑M2/83‑301 (MQ=255)`  
                                                                                                                                                                                                                      `ccactaaagttaccgaggctgcccgtgtggcaaaccaagctcaacagctttccaaattctttgagtaatcaaacaggagaaaCCATTATGATCGTTTCTGACATCGAAGCTAACGCCCTCTTAGAGAGCGTCACTAAGTTCCACTGCGGGGTTATCTACGACTACTCCACCGCTGAGTACGTAAGCTACCGTCCGAGTGACTTCGGTGCGTATCTGGATGCGCTGGAAGCCGAGGTTGCACGAGGCGGTCTTATTGTGTTCCACAACGGTCACAAGTATGACgttcctgcatctgtctctt                                                                                   >  1:241201‑M2/83‑292 (MQ=255)`  
                                                                                                                                                                                                                       `cactaaagttaccgaggctgcccgtgtggcaaaccaagctcaacagctttccaaattctttgagtaatcaaacaggagaaaCCATTATGATCGTTTCTGACATCGAAGCTAACGCCCTCTTAGAGAGCGTCACTAAGTTCCACTGCGGGGTTATCTACGACTACTCCACCGCTGAGTACGTAAGCTACCGTCCGAGTGACTTCGGTGCGTATCTGGATGCGCTGGAAGCCGAGGTTGCACGAGGCGGTCTTATTGTGTTCCACAACGGTTACAAGTATGACGTTCCTGCATTGACCAAACt                                                                                  >  1:97315‑M2/82‑301 (MQ=255)`  
                                                                                                                                                                                                                            `aagttaccgaggctgcccgtgtggcaaatcaagctcaacagctttccaaattctttgagtaatcaaacaggagaaaCCATTATGATCGTTTCTGACATCGAAGCTAACGCCCTCTTAGAGAGCGTCACTAAGTTCCACTGCGGGGTTATCTACGACTACTCCACCGCTGAGTACGTAAGCTACCGTCCGAGTGACTTCGGTTCGTATCTGGATGCGCTGGAAGCCGAGGTTGCACGAGGCGGTCTTATTGTGTTCCACAACGGTCACAAGTATGACGTTCCTGCATTGACCAAACTGGCaa                                                                             >  1:89724‑M2/77‑301 (MQ=255)`  
                                                                                                                                                                                                                            `aagttaccgaggctgcccgtgtggcaaaccaagctcaacagctttccaaattctttgagtaatcaaacaggagaaaTCAATATGATCGTTTCTGACATCGAAGCTAACGCCCTCTTAGAGAGCGTCACTAAGTTCCACTGCGGGGTTATCTACGACTACTCCACCGCTGAGTACGTAAGCTACCGTCCGAGTGACTTCGGTGCGTATCTGGATGCGCTGGAAGCCGAGGTTGCACGAGGCGGTCTTATTGTGTTCCACAACGGTCACAAGTATGACGTTCCTGCATTGACCAAACTGGCaa                                                                             <  1:165129‑M2/225‑1 (MQ=255)`  
                                                                                                                                                                                                                            `aagttaccgaggctgcccgtgtggcaaaccaacctcaacagctttccaaattcttttagtaatcaaacaggagaaaCCATTATGATCGTTTCTGACATCGAAGCTAACGCCCTCTTAGAGAGCGTCACTAAGTTCCACTGCGGGGTTATCTACGACTACTCCACCGCTTATTACGTAAGCTACCGGCCGAGTGACTTCGGTGCGTATCTGGATGCGCTTGAAGCCGAGGTTGCACGAGGCGGTCTTATTGTGTTCCACAACGGTCACAAGTATGACGTTCCTGCATTGACCAAACTGGCaa                                                                             <  1:120676‑M2/225‑1 (MQ=255)`  
                                                                                                                                                                                                                                          `gcccgtgtggcaaaccaagctcaacagctttccaaattctttgagtaatcaaacaggagaaaTCAATATGATCGTTTCTGACATCGAAGCTAACGCCCTCTTAGAGAGCGTCACTAAGTTCCACTGCGGGGTTATCTACGACTACTCCACCGCTGAGTACGTAAGCTACCGTCCGAGTGACTTCGGTGCGTATCTGGATGCGCTGGAAGCCGAGGTTGCACGAGGCGGTCTTATTGTGTTCCACAACGGTCACAAGTATGACgttcctgcattgaccaaactctgtcccttatacacatct                                                               >  1:4408‑M2/63‑282 (MQ=255)`  
                                                                                                                                                                                                                                            `ccgtgtggcaaaccaagctcaacagctttccaaattctttgagtaatcaaacaggagaagTCAATATGATCGTTTCTGACATCGAAGCTAACGCCCTCTTAGAGAGCGTCACTAAGTTCCACTGCGGGGTTATCTACGACTACTCCACCGCTGAGTGCGTAAGCTACCGTCCGAGTGACTTCGGTGCGTATCTGGATGCGCTGGAAGCCGAGGTTGCACGAGGCGGTCTTATTGTGTTCCACAACGGTCACAAGTATGACGTTCCTGCATTGACCAAACTGGCAAAGTTGCAATTGAACCg                                                             <  1:212615‑M2/241‑1 (MQ=255)`  
                                                                                                                                                                                                                                            `ccgtgtggcaaaccaagctcaacagctttccaaattctttgagtaatcaaacaggagaaaTCAATATGATCGTTTCTGACATCGAAGCTAACGCCCTCTTAGAGAGCGTCACTAAGTTCCACTGCGGGGTTATCTACGACTACTCCACCGCTGAGTACGTAAGCTACCGTCCGAGTGACTTCGGTGCGTATCTGGATGCGCTGGAAGCCGAGGTTGCACGAGGCGGTCTTATTGTGTTCCACAACGGTCACAAGTATGACGTTCCTGCATTGACCAAACTGGCAAAGTTGCAATTGTACCg                                                             >  1:254076‑M2/61‑301 (MQ=255)`  
                                                                                                                                                                                                                                                  `ggcaaaccaagctcaacagctttccaaattctttgagtaatcaaacaggagaaaTCAATATGATCGTTTCTGACATCGAAGCTAACGCCCTCTTAGAGAGCGTCACTAAGTTCCACTGCGGGGTTATCTACGACTACTCCACCGCTGAGTACGTAAGCTACCGTCCGAGTGACTTCGGTGCGTATCTGGATGCGCTGGAAGCCGAGGTTGCACGAGGCGGTCTTATTGTGTTCCACAACGGTCACAAGTATGACGTTCCTGCATTGACCACACTGGCAAAGTTGCAATTgaaccgaactgt                                                       >  1:158946‑M2/55‑296 (MQ=255)`  
                                                                                                                                                                                                                                                           `agcgcaacatctttccaaattctttgggtaatcaaacaggagaaaTCATTATGATCGTTTCTGACATCGAAGCTAACGCCCTCTTAGAGAGCGTCACTAAGTTCCACTGCGGTGTTATCTACGACTACTCCACCGCTGAGTACGTAAGCTACCGTCCGAGTGACTTCGGTGCGTATCTGGATGCGCTGGAAGCCGAGGTTGCACGAGGCGGTCTTATTGTGTTCCACAACGGTCACAAGTATGACGTTCCTGCATTGACCAAACTGGCAAAGTTGCAATTGAACCGAGAGTTCCACCTTcc                                              <  1:46814‑M2/256‑1 (MQ=255)`  
                                                                                                                                                                                                                                                                     `ctttccaaattcttagagtaatcaaacaggagaaaTCAAAATGATCGTTTCTGACATCGAAGCTAACGCCCTCTTAGAGAGCGTCACTAAGTTCCACTGCGGGGTTATCTACGACTACTCCACCGCTGAGTACGTAAGCTACCGTCCGAGTGACTTCGGTGCGTATCTGGATGCGCTGGAAGCCGAGGTTGCACGAGGAGGTCTTATTGTGTTCCACAACGGTCACAAGTATGACGTTCCTGCATTGACCAAACTGGCAAAGTTGCAATTGAACCGAGAGTTCCACCTTCCTCGTGAGAAc                                    <  1:331886‑M2/266‑1 (MQ=255)`  
                                                                                                                                                                                                                                                                     `ctcgtgggctcggagatgtgtataagagacagaaaCCATTATGATCGTTTCTGACATCGAAGCTAACGCCCTCTTAGAGAGCGTCACTAAGTTCCACTGCGGGGTTATCTACGACTACTCCACCGCTGAGTACGTAAGCTACCGTCCGAGTGACTTCGGTGCGTATCTGGATGCGCTGGAAGCCGAGGTTGCACGAGGCGGTCTTATTGTGTTCCACAACGGTCACAAGTATGACGTTCCTGCATTGACCAAACTGGCAAAGTTGCAATTGAACCGAGAGTTCCACCTTCCTCGTGAGAAc                                    <  1:115549‑M2/266‑1 (MQ=255)`  
                                                                                                                                                                                                                                                                       `gagatgtgtataagagacagcaaacaggagaaaTCAATATGATCGTTTCTGACATCGAAGCTAACGCCCTCGTAGAGAGCGTCACTAAGTTCCACTGCGGGGTTATCTACGACTACTCCACCGCTGAGTACGTAAGCTACCGTCCGAGTGACTTCGGTGAGTATCTGGATGTGCTGGAAGCCGAGGTTGCACGAGGCGGTCTTATTGTGTTCCACAACGGTCACAAGTATGACGTTCCTGCATTGACCAAACTGGCAAAGTTGCAATTGAACCGAGAGTTCCACCTTCCTCGTGAGAACTg                                  <  1:279169‑M2/268‑1 (MQ=255)`  
                                                                                                                                                                                                                                                                        `tccaaattctttgagtaatcaaacatgagaaaTCAATATGATCGTTTCTGACATCGAAGCTAACGCCCTCTTAGAGAGCGTCACTAAGTTCCACTGCTGGGTTATCTACGAATACTCCACCGCTGAGTACGTAAGCTACCGTCCGAGTGACTTCGGTGCGTATCTGGATGCGCTGGAAGCCGAGGTTGCACGAGGCGGTCTTATTGTGTTCCACAACGGTCACAAGTATGACGTTCCTGCATTGACCAAACTGGCAAAGTTGCAATTGAACCGAGAGTTACACCTTCCTCGTGAGAACTGt                                 >  1:128556‑M2/33‑301 (MQ=255)`  
                                                                                                                                                                                                                                                                        `tccaaattctttgagtaatcaaacaggagaaaTCAATATGATCGTTTCTGACATCGAAGCTAACGCCCTCTTAGAGAGCGTCACTAAGTTCCACTGCGGGGTTATCTACGACTACTCCACCGCTGAGTACGTAAGCTACCGTCCGAGTGACTTCGGTGCGTATCTGGATGCGCTGGAAGCCGAGGTTGCACGAGGCGGTCTTATTGTGTTCCACAACGGTCACAAGTATTACGTTCCTGCATTGACCAAACTGGCAAAGTTGCAATTGAACCGAGAGTTCCACCTTCCTCGTGCGAACTGt                                 >  1:284474‑M2/33‑301 (MQ=255)`  
                                                                                                                                                                                                                                                                             `attctttgagtaatcaaacaggagaaaTCAATATGATCGTTTCTGACATCGAAGCTAACGCCCTCTTAGAGAGCGACACTAAGTTCCACTGCGGGGTTATCTACGACTACTCCAACGCTGAGTACGAAAGCTACCGGCCGAGTGACTTCGGTGCGTATCTGGCTGCGCTGGAAACCGAGGCAGCACGAGGCGGTCTTATTGTGTTCCACAACGGTCACAAGTATGACGCTCCTACATTGACCACACTGGCAAAGTTGCAATCGAACCGAATGCTCCACCTTCCTCGTGCGAACTGACTTGa                            >  1:93482‑M2/28‑301 (MQ=255)`  
                                                                                                                                                                                                                                                                              `ttctttgagtaatcaaacaggagaaaCCATTATGATCGTTTCTGACATCGAAGCTAACGCCCTCTTAGAGAGCGTCACTAAGTTCCACTGCGGGGTTATCTACGACTACTCCACCGCTGAGTACGTAAGCTACCGTCCGAGTGACTTCGGTGCGTATCTGGATGCGCTGGAAGCCGAGGTTGCACGAGGCGGTCTTATTGTGTTCCACAACGGTCACAAGTATGACGTTCCTGCATTGACCAAACTGGCAAAGTTGCAATTGAACCGAGAGTTCcaccttcctcgtgagctgtctcttata                           >  1:275941‑M2/27‑289 (MQ=255)`  
                                                                                                                                                                                                                                                                               `tctttgagtaatcaaacaggagaaaCCATTATGATCGTTTCTGACATCGAAGCTAACGCCCTCTTAGAGAGCGTCACTAAGTTCCACTGCGGGGTTATCTACGACTACTCCACCGCTGAGTACGTAAGCTACCGTCCGAGTGACTTCGGTGCGTATCTGGATGCGCTGGAAGCCGAGGTTGCACGAGGAGGTCTTATTGTGTTCCACAACGGTCACAAGTATGACGTTCCTGCATTGACCAAACTGGCAAAGTTGCAATTGAACCGAGAGTTCCACCTTCCTCGTGAGAACTGTATTGaca                          >  1:290002‑M2/26‑301 (MQ=255)`  
                                                                                                                                                                                                                                                                                 `ctcggagatgtgtataagagacaGCAATATGATCGTTTCTGACATCGAAGCTAACGCCCTCTTAGAGAGCGTCACTAAGTTCCACTGCGGGGTTATCTACGACTACTCCACCGCTGAGTACGTAAGCTACCGTCCGAGTGACTTCGGTGCGTATCTGGATGCGCTGGAAGCCGAGGTTGCACGAGGCGGTCTTATTGTGTTCCACAACGGTCACAAGTATGACGTTCCTGCATTGACCAAACTGGCAAAGTTGCAATTGAACCGAGAGTTCCACCTTCCTCGTGAGAACTGTATTGACAcc                        <  1:216253‑M2/278‑1 (MQ=255)`  
                                                                                                                                                                                                                                                                                        `aatcaaacaggagaaaTCAATATGATCGTTTCTGACATCGAAGCTAACGCCCTCTTAGAGAGCGTCACTAAGTTCCACTGCGGGGTTATCTACGACTACTCCACCGCTGAGTACGTAAGCTACCGTCCGAGTGACTTCGGTGCGTATCTGGATGCGCTGGAAGCCGAGGTTGCACGAGGCGGTCTTATTGTGTTCCACAACGGTCACAAGTATGACGTTCCTGCATTGACCAAACTGGCAAAGTTGCAATTGAACCGAGAGTTCCACCTTCCTCGTGAGAACTGTATTGACACCCTTgtgt                 <  1:19121‑M2/285‑1 (MQ=255)`  
                                                                                                                                                                                                                                                                                          `ataagagacagaaaCCATTATGATCGTTTCTGACATCGAAGCTAACGCCCTCTTAGAGAGCGTCACTAAGTTCCACTGCGGGGTTATCTACGACTACTCCACCGCTGAGTACGTAAGCTACCGTCCGAGTGACTTCGGTGCGTATCTGGATGCGCTGGAAGCCGAGGTTGCACGAGGCGGTCTTATTGTGTTCCACAACGGTCACAAGTATGACGTTCCTGCATTGACCAAACTGGCAAAGTTGCAATTGAACCGAGAGTTCCACCTTCCTCGTGAGAACTGTATTGACACCCTTGtgttg               <  1:219515‑M2/287‑1 (MQ=255)`  
                                                                                                                                                                                                                                                                                                 `ggagaaaTCAATATGATCGTTTCTGACATCGAAGCTAACGCCCTCTTAGAGAGCGTCACTAAGTTCCACTGCTGGGTTATCTACGACTACTCCACCGCTGAGTACGTAAGCTACCGTCCGAGTGACTTCGGTGCGTATCTGGATGCGCTTGAAGCCGCGGTTGCACGAGGCGGTCTTATTGTGTTCCACAACGGTCACAAGTATGACGTTCCTGCATTGACCAAACTGGCAAAGTTGCAATTGAACCGAGAGTTCCACCTTCCTCGTGAGAACTGTATTGACCCCCTTGTGTTGTCACGtt        >  1:340911‑M2/8‑301 (MQ=255)`  
                                                                                                                                                                                                                                                                                                  `gagaaaCCATTATGATCGTTTCTGACATCGAAGCTAACGCCCTCTTAGAGAGCGTCACTAAGTTCCACTGCGGGGTTATCTACGACTACTCCACCGCTGAGTACGTAAGCTACCGTCCGAGTGACTTCGGTGCGTATCTGGATGCGCTGGAAGCCGAGGTTGCACGAGGCGGTCTTATTGTGTTCCACAACGGTCACAAGTATGACGTTCCTGCATTGACCAAACTGGCAAAGTTGCAATTGAACCGAGAGTTCCACCTTCCTCGTGAGAACTGTATTGACACCCTTGTGTTGTCACGttt       >  1:40012‑M2/7‑301 (MQ=255)`  
                                                                                                                                                                                                                                                                                                     `aaaCCATTATGATCGTTTCTGACATCGAAGCTAACGCCCTATTAGAGAGCGTCACTAAGTTCCACTGCGGGGTTATCTACGACTACTCCACCGCTGAGTACGTAAGCTACCGTCCGAGTGACTTCGGTGCGTATCTGGATGCGCTGGAAGCCGAGGTTGCACGAGGCGGTCTTATTGTGTTCCACAACGGTCACAAGTATGACGTTCCTGCATTGACCAAACTGGCAAAGTTGCAATTGAACCGAGAGTTCCACCTTCCTCGTGAGAACTGTATTGACACCCTTGTTTTTTCACGTTTGAt    >  1:23624‑M2/4‑301 (MQ=255)`  
                                                                                                                                                                                                                                                                                                       `aTCAATATGATCGTTTCTGACATCGAAGCTAACGCCCTCTTAGAGGGCGTCACTAAGTTCCACTGCGGGGTTATCTACGACTACTCCACCGCTGAGGACGTAAGCTACAGTCAGAGTGACTTCGGTGCGTATCTGGATGCGCTGGAAGCCGAGGTTGCACGAGGCGGTCTTATTGTGTTCCACAACGGTCACAAGTATGACGTTCCTGAATTGAACAAACTGGCAAAGTGGCAATTGAACCGAGAGTTGGACCTGCATCGTGAGAACGGTATTGACACCCTTGTGTTGTGAcgtttgtttt  >  1:315967‑M2/2‑300 (MQ=255)`  
                                                                                                                                                                                                                                                                                                       `aTCAATATGATCGTTTCTGACATCGAAGCTAACGCCCTCTTAGAGAGCGTCACTAAGTTCCACTGCGGGGTTATCTACGACTACTCCACCGCTGAGTACGTAAGCTACCGTCCGAGTGACTTCGGTGCGTATCTGGATGCGCTGGAAGCCGAGGTTGCACGAGGCGGTCTTATTGTGTTCCACAACGGTCACAAGTATGACGTTCCTGCATTGACCAAACTGGCAAAGTTGCAATTGAACCGAGAGTTCCACCTTCCTCGTGAGAACTGTATTGACACCCTTGTGTTGTCACGTTTGattt  >  1:247971‑M2/2‑300 (MQ=255)`  
                                                                                                                                                                                                                                                                                                       `aTCAATATGATCGTTTCTGACATCCAAGCTAACGCCCTCTTAGAGAGCGTCACTCAGTTCCACTGCGGGGTTATCTACGACTACTCCACCGCTGAGTACGTAAGCTACCGTCCGAGTGACTTCGGTGCGTATCTGGATGCGCTGGAAGCCGAGGTTGCACGAGGCGGTCTTATTGTGTTCCACAACGGTCACAAGTATGACGATCCTGCATTGACCAAACTGGCAAAGTTGCAATTGAATCGAGAGTTCCACCTTCCTCGTGAGAACTGTATTGACACCCTTGTGTTGTCACGTTTGATTc  <  1:345580‑M2/300‑1 (MQ=255)`  

                                                                                                                                                                                                                                                                                                        `|`                                                                                                                                                                                                                                                                                                             
`GACAAGACAGACTGGCTGGAAGAAACCAGAAGTTACCAAGCTGAGTCATAAGGTGTTCGATAAGGACACTATGACCCACATCAAGGCTGGTGATTGGGTTAAGGTTGACATGGGAGTTGTTGGTGGATACGGCTACGTCCGCTCAGTTAGTGGCAAATATGCACAAGTGTCATACATCACAGGTGTTACTCCACGCGGTGCAATCGTTGCCGATAAGACCAACATGATTCACACAGGTTTCTTGACAGTTGTTTCATATGAAGAGATTGTTAAGTCACGATAATCAATAGGAGAAATCAATATGATCGTTTCTGACATCGAAGCTAACGCCCTCTTAGAGAGCGTCACTAAGTTCCACTGCGGGGTTATCTACGACTACTCCACCGCTGAGTACGTAAGCTACCGTCCGAGTGACTTCGGTGCGTATCTGGATGCGCTGGAAGCCGAGGTTGCACGAGGCGGTCTTATTGTGTTCCACAACGGTCACAAGTATGACGTTCCTGCATTGACCAAACTGGCAAAGTTGCAATTGAACCGAGAGTTCCACCTTCCTCGTGAGAACTGTATTGACACCCTTGTGTTGTCACGTTTGATTC  >  NC_001604.1/14052‑14647`

  

Alignment Legend

Aligned base mismatch/match (shaded by quality score): `ATCG/ATCG` `< 3 ≤` `ATCG/ATCG` `< 11 ≤` `ATCG/ATCG` `< 24 ≤` `ATCG/ATCG` `< 36 ≤` `ATCG/ATCG` `< 38 ≤` `ATCG/ATCG`

Unaligned base: `atcg`    Masked matching base: `atcg`    Alignment gap: `‑`    Deleted base: `‑`
